# What are the long‐term holistic health consequences of COVID‐19 among survivors? An umbrella systematic review

**DOI:** 10.1002/jmv.28086

**Published:** 2022-09-03

**Authors:** Catherine Paterson, Deborah Davis, Michael Roche, Bernie Bissett, Cara Roberts, Murray Turner, Emma Baldock, Imogen Mitchell

**Affiliations:** ^1^ Faculty of Health University of Canberra Bruce Australian Capital Territory Australia; ^2^ Prehabilitation, Activity, Cancer, Exercise and Survivorship (PACES) Research Group University of Canberra Bruce Australian Capital Territory Australia; ^3^ School of Nursing, Midwifery and Public Health University of Canberra Bruce Australian Capital Territory Australia; ^4^ Canberra Health Services & ACT Health SYNERGY Nursing & Midwifery Research Centre Canberra Australian Capital Territory Australia; ^5^ School of Nursing, Midwifery & Paramedic Practice Robert Gordon University Aberdeen Scotland UK; ^6^ Schoool of Nursing University of Technology Sydney Ultimo New South Wales Australia; ^7^ Health Care Consumers' Association of the Australian Capitial Territory Australia; ^8^ Australian National University Canberra Australian Capital Territory Australia; ^9^ Canberra Health Services Canberra Australian Capital Territory Australia

**Keywords:** coronavirus, epidemiology, pandemics, SARS coronavirus, virus classification

## Abstract

Many people who have survived COVID‐19 have experienced negative persistent impacts on health. Impacts on health have included persistent respiratory symptoms, decreased quality of life, fatigue, impaired functional capacity, memory deficits, psychological impacts, and difficulties in returning to paid employment. Evidence is yet to be pooled to inform future directions in research and practice, to determine the physical, psychological, social, and spiritual impacts of the illness which extend beyond the acute phase of COVID‐19 survivors. This umbrella review (review of systematic reviews) critically synthesized physical (including abnormal laboratory parameters), psychological, social, and spiritual impacts which extended beyond the acute phase of COVID‐19 survivors. The search strategy was based on the sample, phenomena of interest, design, evaluation, research model and all publications were double screened independently by four review authors for the eligibility criteria. Data extraction and quality assessment were conducted in parallel independently. Eighteen systematic reviews were included, which represented a total of 493 publications. Sample sizes ranged from *n* = 15 to *n* = 44 799 with a total of *n* = 295 455 participants. There was incomplete reporting of several significant data points including the description of the severe acute respiratory syndrome coronavirus 2 variant, COVID‐19 treatments, and key clinical and demographic data. A number of physical, psychological, and social impacts were identified for individuals grappling with post‐COVID condition. The long term sequalae of acute COVID‐19 and size of the problem is only beginning to emerge. Further investigation is needed to ensure that those affected by post‐COVID condition have their informational, spiritual, psychological, social, and physical needs met in the future.

## 1 INTRODUCTION

Severe acute respiratory syndrome (SARS) coronavirus 1 is a strain of coronavirus that causes the SARS, the respiratory illness responsible for the 2002–2004 SARS outbreak. The severe acute respiratory syndrome coronavirus 2 (SARS‐CoV‐2) was detected in China in December 2019.^1^ Since this time there have been 465 million confirmed cases and 6 million deaths from SARS‐CoV‐2.^2^ During this pandemic, humanity has observed an unprecedented effort from health and scientific communities to diagnose, treat and prevent COVID‐19, however, the long‐term physical and psychological sequelae of this disease among survivors are yet to be fully understood.^3^ Within the published literature, defining the post‐COVID condition is problematic because of a range of different terms, which are not standardized or consistent. Terms such as long COVID‐19,^4^ postacute COVID‐19 syndrome,^3^ long‐term effects of COVID‐19,^5^ long‐haulers,^6^ and persistent COVID‐19 symptoms^7^ have all been used to describe persistent signs and symptoms, or physiological measurements which have not returned to normal levels.^8^ Further complicating these definitions, the “timeframe” employed to describe residual signs and symptoms is highly variable and the spectrum of long‐term consequences is broad, encompassing the physical, psychological, social, and spiritual dimensions of health^9^ among COVID‐19 survivors. The timeframes specified to define persistent side‐effects of COVID‐19 include: (a) an illness in which individuals who have recovered from COVID‐19 continue to experience unusual symptoms longer than expected^10^; (b) persistent symptoms 2 weeks following COVID‐19 recovery^5^; and (c) symptoms that have continued for more than 3 months post COVID‐19.^11^ Moreover, the National Institute for Health and Care Excellence (NICE) distinguishes between ongoing symptomatic COVID‐19 which lasts between 4 and 12 weeks, and post COVID‐19 syndrome which is sustained beyond 12 weeks.^12^ Therefore, given the heterogeneity within the existing literature, this review of post‐COVID conditions will use an inclusive classification of all changes in physical, psychological, social, and spiritual domains of health irrespective of the duration following the initial acute disease episode of COVID‐19, which is inclusive of the first 4 weeks.^12^ This holistic approach to defining the subsequent impacts of COVID‐19 on health could guide the future provision and design of multidisciplinary services for those affected following acute COVID‐19. Developing personalized services by understanding an individual's informational, spiritual, psychological, social, and physical needs during follow‐up phases would improve the survivor experience. It would also include issues of health promotion and prevention and COVID‐19 individualized rehabilitation.^13^

Numerous systematic reviews have been conducted to understand the longer‐term impact of COVID‐19 on health and well‐being among survivors.^5^, ^11^, ^14^, ^15^, ^16^, ^17^, ^18^, ^19^ This evidence reveals that globally many people who have survived COVID‐19 have experienced negative persistent impacts on health, including financial implications,^11^ however, the exact numbers of those affected remain unknown.^18^ Impacts on health have included persistent respiratory symptoms, decreased quality of life, fatigue, impaired functional capacity, memory deficits, psychological impacts and difficulties in returning to paid employment.^11^ Despite the many systematic reviews conducted on the topic, the evidence is yet to be pooled for the purpose of informing future clinical trials, clinical guidelines, and policy for future multidisciplinary team clinical service design to address the holistic person‐centered needs of COVID‐19 survivors. Therefore, the aim of this study is to present an umbrella systematic review (a review of reviews) to summarize the evidence, appraise its quality, and combine relevant data to provide clinical decision makers with the evidence they need for targeted interventions to improve holistic health outcomes for people affected by COVID‐19. Umbrella systematic reviews enable a systematic approach to appraise the evidence on an entire topic in relation to addressing the following research question:

- Among COVID‐19 survivors, what are the physical, psychological, social, and spiritual impacts of the illness which extend beyond the acute phase?

## 2 MATERIALS AND METHODS

### 2.1 Study design

The Joanna Briggs Institute (JBI) umbrella review method^20^ was employed to provide an overall examination of the body of evidence that was available in relation to the physical (including abnormal laboratory parameters), psychological, social, and spiritual impacts which extended beyond the acute phase of COVID‐19 survivors. The key features of this review design are that it: (1) compiled evidence from multiple research syntheses that are qualitative and/or quantitative in nature, (2) included reviews that are based upon empirical studies rather than theoretical speculations or opinions, and (3) summarized evidence from existing reviews without resynthesis of the primary studies. This review has been reported according to the Preferred Reporting Items for Systematic Reviews and Meta‐Analyses (PRISMA) 2020 statement guidelines.^21^

### 2.2 Types of participants

This umbrella review included multiple participants with diverse clinical and demographic characteristics across the entire lifespan who were affected by COVID‐19. Wide inclusion was important because it has not yet been established how age, gender, pregnancy, ethnicity, existing comorbidities, viral load, or invasive medical interventions affect the risk of developing long‐term effects of COVID‐19 among survivors.

### 2.3 Types of reviews

All qualitative, quantitative, and mixed methods reviews (systematic review, meta‐analysis, narrative review, descriptive review, scoping review, qualitative review, realist review, critical review, literature review, mixed methods reviews, qualitative evidence synthesis, rapid review, review of reviews) were included irrespective of review design. Reviews were excluded if they did not describe the search strategy, inclusion criteria, and quality assessment methods. All reviews where the primary aim/research question for the review did not describe the physical, psychological, social, and spiritual impacts of COVID‐19 beyond the acute phase were excluded. Reviews in languages other than English were counted but not read or evaluated. Reviews not based on primary empirical studies were also excluded.

### 2.4 Phenomena of interest/outcomes

The main phenomenon of interest was the experience of physical, psychological, social, and spiritual impacts extending beyond the acute phase of COVID‐19 among survivors.

### 2.5 Context setting

The context included diverse geographical locations, a wide range of cultural factors, and different health care settings (acute, primary, and community health care), including wider clinical and demographic profiles of COVID‐19 survivors.

### 2.6 Search strategy

The search strategy was based on the sample, phenomena of interest, design, evaluation, research (SPIDER) model.^22^ The SPIDER model is a tool developed for research questions and consists of five domains of interest, namely:

•Sample (S): People affected by long‐term consequences of COVID‐19.

•Phenomena of Interest (PI): The physical, psychological, social, and spiritual impacts on health among COVID‐19 survivors.

•Design (D): All qualitative, quantitative, and mixed methods reviews.

•Evaluation (E): N/A.

•Research (R): Systematic reviews.

In this context, “evaluation” was not applied in the string due to the nature of the umbrella study. A comprehensive search was conducted in Medline, CINAHL, Scopus, and PsycINFO databases from inception to October 2021. Search terms included variations of MeSH terms and keywords to increase the sensitivity and inclusiveness of searches. See Supporting Information: Table 1 for a full record of database searches. All records were managed using Endnote X20 and uploaded to Covidence systematic review software for deduplication of records and the study selection process. A preselection eligibility criterion was applied to all records.

### 2.7 Systematic review selection

All publications (titles and abstracts) were double screened independently by four review authors to promote consistency and reliability in the application of the eligibility criteria. Articles that met the inclusion criteria were retrieved in full text and double screened with any disagreements resolved by discussion.

### 2.8 Critical appraisal systematic reviews and research synthesis

Systematic reviews that were eligible for inclusion were assessed for methodological quality (critical appraisal) using the JBI tool^23^ performed by two reviewers and cross‐checked together. Each criterion was scored as being “met,” “not met” “unclear” or “not applicable,” see Table 1.

**Table 1:** Critical appraisal checklist for systematic reviews and research syntheses

Is the review question clearly and explicitly stated?
Were the inclusion criteria appropriate for the review question?
Was the search strategy appropriate?
Were the sources and resources used to search for studies adequate?
Were the criteria for appraising studies appropriate?
Was critical appraisal conducted by two or more reviewers independently?
Were the methods used to combine studies appropriate?
Was the likelihood of publication bias assessed?
Were recommendations for policy and/or practice supported by the reported data?
Were the specific directives for new research appropriate?

*Note*: Scored as being “met,” “not met” “unclear” or “not applicable.”

### 2.9 Data extraction

Data extraction was cross‐referenced by two reviewers using templates guided by JBI.^20^ Key information was extracted from each systematic review including: (1) citation details, (2) objectives of the included review, (3) type of review, (4) participant details, (5) setting and context, (6) number of databases sourced and searched, (7) date range of database searching, (8) publication date range of studies included in the review, (9) number of studies, types of studies and country of origin of studies included in each review, (10) instrument used to appraise the primary studies and the rating of their quality, (11) outcomes reported that are relevant to the umbrella review question, (12) method of synthesis/analysis employed to synthesize the evidence, and (13), comments or notes the umbrella review authors may have regarding any included study.

### 2.10 Data synthesis

A metalevel narrative synthesis^20^, ^23^ of the findings across the included reviews was structured around (1) the type of reviews (qualitative or quantitative), (2) the target population's characteristics, and (3) outcomes related to impacts on holistic health postacute COVID‐19. Specifically, this involved data reduction (subgroup classification by domain of health, with results tabulated), data comparison (identifying patterns and themes through clustering and counting and making contrasts and comparisons), and conclusion drawing and verification (synthesis of subgroup analysis to inform a comprehensive understanding of the topic, verified with the primary source data for accuracy).

## 3 RESULTS

**Figure 1:**
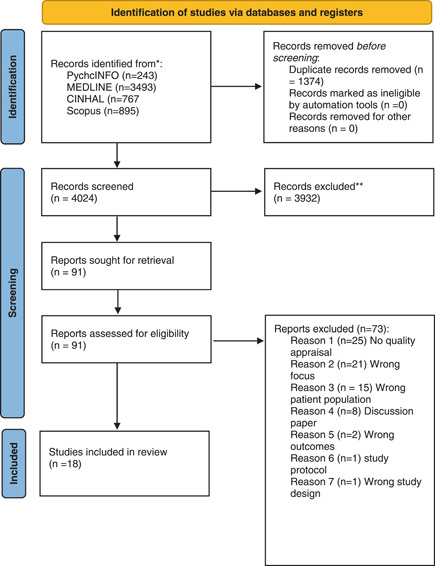
Preferred reporting items for systematic reviews and meta‐analyses diagram^21^

**Figure 2:**
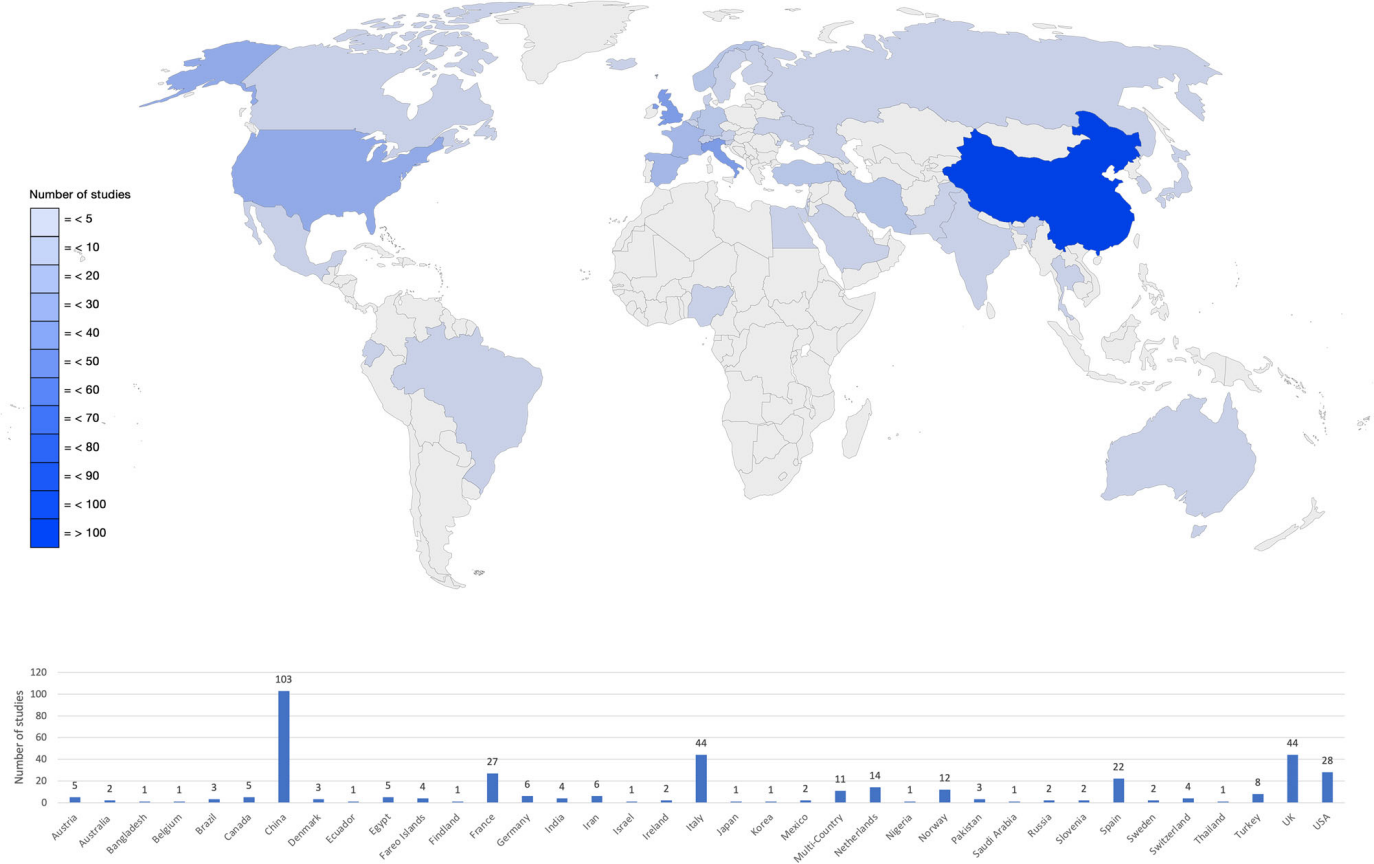
Worldmap of the distribution of primary studies

A total of 18 systematic reviews were included, which represented a total of 493 articles. Figure 1 presents the PRIMSA flowchart of the literature search and selection process. A range of study designs were included: retrospective chart reviews (*n* = 18), case series (*n* = 2), cohort studies (*n* = 129), point prevalence study (*n* = 1), prospective longitudinal studies (*n* = 60), cross‐sectional (*n* = 185), case‐control (*n* = 6), qualitative (*n* = 3), randomized controlled trial (*n* = 1), mixed methods (*n* = 1), case study (*n* = 6) and three systematic reviews^5^, ^24^, ^25^ that did not report primary study designs. There were a range of countries represented within the systematic reviews (Figure 2), but the geographical location of included studies was not reported in four systematic reviews.^24^, ^25^, ^26^, ^27^ There is a lack of research with non‐WEIRD (Westernised, Educated, Industrialized, Rich, Democratic)^28^ populations represented. Sample sizes ranged from *n* = 15 to *n* = 44 799 with a total of *n* = 295 455 participants represented in this umbrella review. Two reviews did not report participant numbers.^24^, ^27^ Across all the included systematic reviews, there was incomplete reporting for demographic variables such as age, gender, pre‐existing comorbidities, vaccination status and whether participants had a hospital admission or were admitted to the intensive care unit (ICU) due to COVID‐19. This incomplete data is a limitation as all these factors are likely to impact on the experience of post‐COVID condition. Other important omissions included a lack of reporting and consideration in study designs to account for: (1) different COVID‐19 variants, (2) COVID‐19 treatments, requirement for supplemental oxygen, ventilation, and so forth, (3) lack of healthy age‐matched controls, (4) absent control for change over time, (5) racial differences, (6) gender, (7) pregnancy, (8) frequency, severity, and burden of symptoms at the time of disease onset, (9) influence of biological factors (such as immune, inflammatory, genetic and metabolic function, black and white fungus), and (10) geographical differences, and (11) influence of pre‐existing mental health disorders, all of which may contribute directly to the experience and reporting of long‐COVID sequalae. These are important shortcomings in the interpretation of the existing evidence across the suite of included systematic reviews (Table 2). Overall, the methodological quality of the included systematic reviews was of medium to high quality (Table 3).

**Table 2:** Table of included review characteristics

Author and year country	Objective of the included review	Participant details	Number of databases sourced and searched	Data range of the searches	Number of studies
Aiyegbusi et al. (2021) UK	To identify symptom prevalence, complications and management of long COVID.	Sample size: Not reported. Age: Not reported. Gender: Not reported. Comorbidities: Not reported. Hospital admission: Not reported. ICU: Not reported. Follow‐up timepoint: 19 articles described ongoing symptomatic COVID‐19 (symptoms lasting 4–12 weeks) and eight studies reported post‐COVID‐19 syndrome (symptoms persisting beyond 12 weeks). Socioeconomic status: Not reported.	PubMed, EMBASE, MedRxiv, and BioRxiv.	Searched the living systematic review database on February 8, 2021.	*n* = 27
Alnefeesi et al. (2021) Canada	To explore the impact of COVID‐19 on cognition during the acute and recovery phases of the disease.	Sample sizes: *n* = 644. Age: mean age 69 years (SD 7.9). Gender: Females: ranged from 11 (37%) to 83 (38%). Comorbidities: *n* = 72 of the overall sample had premorbid dementia. Hospital admission: Not reported. ICU: Not reported. Follow‐up timepoint: Not reported. Socioeconomic status: Not reported.	CINAHL Plus, MEDLINE, EMBASE, APA PsycINFO.	2019 to August 26, 2020.	*n* = 7
Deng et al. (2020) Canada	To explore the impact of the pandemic on COVID‐19 patients' mental health.	Sample sizes: *n* = 5153. Age: mean age ranged from 35.9 (SD 11.9) to 72.6 (SD 12.1) years. Gender: Median male representation of 49% (range 25%–62%). Comorbidities: Not reported. Hospital admission: Not reported. ICU: Not reported. Follow‐up timepoint: Not reported. Socioeconomic status: Not reported.	MEDLINE, EMBASE, PubMed, Web of Science, CINAHL. Chinese databases: Wanfang Data, Wanfang Med Online, China National Knowledge Infrastructure (CNKI), Chongqing VIP Information.	2019 to August 18, 2020.	*n* = 31
Dong et al. (2021) China	To explore the characteristics and related factors of psychological problems among COVID‐19 patients.	Sample sizes: *n* = 8587. Age: Not reported. Gender: Female: *n* = 2744 Male: *n* = 2588. Comorbidities: Not reported. Hospital admission: Twenty‐five studies (56.8%) investigated COVID‐19 patients with no distinguishing feature; two (4.5%) included discharged COVID‐19 patients; two (4.5%) included severe COVID‐19 patients; six (13.6%) studies included mild or clinically stable COVID‐19 patients; nine (20.5%) included suspected COVID‐19 patients. Socioeconomic status: No study set in a low‐middle income country.	PubMed, Embase, PsycInfo, Wanfang Data, Chongqing VIP, Sinomed, and CNKI.	January 1, 2020 to October 7, 2020.	*n* = 44
Fernandez‐de‐las‐Penas et al. (2021) Spain	To synthesize the prevalence of post‐COVID pain symptoms of musculoskeletal origin in hospitalized/nonhospitalized patients recovered from SARS‐CoV‐2 infection.	Sample size: *n* = 25 709. Age: 47.25 (SD: 15.8 years). Gender: Female: 42.74%. Comorbidities: Hypertension (23.8%, 95% CI 17.6%–31.2%) and obesity (22.2%, 95% CI 13.7%–34.0%) were the comorbidities more prevalent. Pre‐existing comorbidities were, in general, more prevalent in hospitalized patients than in nonhospitalized patients, being statistically significant for obesity, hypertension, diabetes, heart and kidney diseases (all, *p* < 0.01). Hospital admission: ICU: *n* = 22 studies included hospitalized patients and *n* = 12 nonhospitalised samples. Socioeconomic status: No study set in a low‐middle income country.	MEDLINE, CINAHL, PubMed, EMBASE, Web of Science.	Search up to May 1, 2021.	*n* = 27
Fernandez‐de‐las‐Penas et al. (2021) Spain	To identify the prevalence of post‐COVID‐19 symptoms among hospitalized and nonhospitalized patients and identify the time course.	Sample sizes: *n* = 15 577 Age: 47.8 years (SD 16.6) Gender: Female: 52.26% Comorbidities: 50% of the total sample exhibited at least one pre‐existing comorbidity (one: 26.3%, 95% CI 25.3%–28.0%; two: 17.6%, 95% CI 15.1%–20.5%; ≥3: 25.6%, 95% CI 11.4%–47.8%) with hypertension (22.9%, 95% CI 16.2%–31.5%) and obesity (22.2%, 95% CI 13.9%–33.5%) being the most prevalent. Hospital admission: The mean length of hospital stay due to SARS‐CoV‐2 infection was 12.5 days (SD 6.8). ICU: From those hospitalized, 402 patients (8%) required ICU admission (mean stay: 15 ± 14.6 days). Socioeconomic status: No study set in a low‐middle income country.	MEDLINE, CINAHL, PubMed, EMBASE, Web of Science.	Searched to March 15, 2021.	*n* = 29
Fernandez‐de‐las‐Penas et al. (2021) Spain	To explore the time course of headache from infection to different post‐COVID follow‐up periods and differentiating whether patients were hospitalized or not.	Sample sizes: *n* = 28 438. Age: 46.6 (SD 17.45) years. Gender: Female: *n* = 12 307. Comorbidities: Not reported. Hospital admission: 30 days (*n* = 11, five hospitalized and six non/hospitalized), 60 days (*n* = 9, four hospitalized and five nonhospitalized), 90 days (*n* = 11, six hospitalized and five nonhospitalized), and ≥180 days (*n* = 13, five hospitalized and eight nonhospitalized) after hospital discharge or symptoms' onset. Socioeconomic status: No study set in a low‐middle income country.	MEDLINE, CINAHL, PubMed, EMBASE, Web of Science.	Searched up to May 31, 2021.	*n* = 28
Iqbal et al. (2021) UK	To synthesis evidence to describe the clinical features of acute and chronic post‐COVID syndrome, and to identify predictor variables.	Sample size: *n* = 12 974. Age: Mean age range: 4.6–70 years. Mean age <10 years 1/43, <20 0/43, <30 0, <40 4/43, <50 15/43, <60 13/43, <70 8/43, <80 2/43. Gender: Females % range: 23–85, <30% 1/43, <40% 11/43, <50% 11/43, <60% 11/43, <70% 3/43, <80% 3/43, <90% 3/43. Comorbidities: Reported in the majority of studies hypertension 20/43, asthma, 25/43, diabetes 19/43 CKD 5/43 and not reported 17/43. Hospital admission: Not reported. ICU: Not reported. Socioeconomic status: No study set in a low‐middle income country.	Ovid in Medline, EMBASE, health management information consortium (HMIC), and PsycINFO.	Searched to March 06, 2021 to from date not reported.	*n* = 45
Long et al. (2021) China	To describe the residual symptoms and pulmonary function tests of discharged COVID‐19 patients (including those discharged from ICU) in the postacute phase.	Sample size: *n* = 4478. Age: Mean ages generally between 50 and 60 years old. Gender: Male: *n* = 2309 (51.56%). Comorbidities: Not reported. Hospital admission: Not reported. ICU: Not reported. Socioeconomic status: Not reported and no study set in a low‐middle income country.	PubMed, Embase, Web of Science, and WHO COVID‐19 Database.	January 1, 2020 to February 23, 2021.	*n* = 16
Lopez‐Leon et al. (2021) USA	To identify studies assessing long‐term effects of COVID‐19 and estimate the prevalence of each symptom, sign, or laboratory parameter of patients at a post‐COVID‐19 stage.	Sample size: *n* = 47 910. Age: Range: 17–87 years. Gender: Male: Ranged from 24.6% to 87.5% of samples. Comorbidities: Not reported. Hospital admission: 6/15 (40%) studies recorded hospital admission. ICU: 4/15 (27%) of studies recorded ICU admission. Socioeconomic status: Not reported.	LitCOVID (PubMed and Medline), Embase.	Published before January 1, 2021.	*n* = 15
Michelen et al. (2021) UK	To regularly synthesize evidence on long COVID characteristics, to help inform clinical management, rehabilitation strategies, and interventional studies to improve long‐term outcomes.	Sample size: *n* = 10 951. Age: Range: 9 months to 93‐year‐old (*n* = 4/39 or 4% of studies included children). Gender: Females: *n* = 5206/10 951 (48%). Comorbidities: Reported in the majority of studies (85%, 33/39), with hypertension and diabetes most commonly documented. Hospital admission: *n* = 8520/10 951 (78%) hospitalized during the acute phase. ICU: *n* = 22/39 (56%) of studies included people requiring ICU admission during the acute phase. Socioeconomic status: No study set in a low‐middle income country.	Medline and CINAHL (EBSCO), Global Health (OVID), WHO Global Research Database on COVID‐19, LitCovid.	January 1, 2020 to March 17, 2021.	*n* = 39
Nasserie et al. (2021) USA	To conduct a systematic review of studies examining the frequency and variety of persistent symptoms after COVID‐19 infection.	Sample size: *n* = 9751. Age: Reported mean or median ages <60 years: 30/45 (67%) ≤50 years: 14/45 (31%). Gender: Male: *n* = 5266 (54%). Comorbidities: Most frequently reported were diabetes and hypertension. Diabetes: 34/45; 75% (median frequency, 16.6%; IQR, 10.0%–23.0%). *Hypertension*: 32/45; 71% (median frequency, 35.0%; IQR, 21.8%–41.0%). Hospital admission: Inpatients: 33/45 (73%) of studies. Outpatients: 2/45 (4%) of studies. Combination: 10/45 (22%) with inpatients ranging from 23% to 80%. ICU: Of those studies that indicated ICU admissions the range was 1.7%–100% of patients. Socioeconomic status: Not reported.	PubMed and Web of Science.	January 1, 2020 to March 11, 2021.	*n* = 45
Pizarro‐Pennarolli et al. (2021) Chile	To understand the impact of COVID‐19 on ADL performance of adult patients and to describe the common scales used to assess performance of ADL on patients post‐COVID‐19.	Sample size: *n* = 1465. Age: Median was 68.98 years (±8.29). Gender: Female: 16/1465 (48.9%). Comorbidities: 4/9 (44%) of studies mentioned comorbidities. Of those, two studies specifically report hypertension and diabetes mellitus. Hospital admission: 1190/1465 (81.2%). ICU: Not reported. Socioeconomic status: Not reported.	Embase, Cochrane Library, CINAHL, Web of Science and PubMed/MEDLINE.	December 1, 2019 to September 10, 2020.	*n* = 9
Sanchez‐Ramirez et al. (2021) Canada	To explore post‐COVID‐19 effects on patients chest computed tomography, lung function, respiratory symptoms, fatigue, functional capacity, health‐related quality of life (HRQoL), and the ability to return to work beyond 3 months post infection.	Sample size: *n* = 5323. Age: Mean age 55.2 (±8.1) years. Gender: Male: 56% Comorbidities: Not reported. Hospital admission: Severe illness due to COVID‐19, defined as the presence of pneumonia, serious or critical illness, need for hospitalization, ICU care, use of supplemental oxygen, and so forth, was reported in ∼36% of the cohort at baseline. ICU: 8/24 (33%) reported data on ICU patients. Socioeconomic status: Not reported.	PubMed, Web of Science, Ovid MEDLINE.	Not reported. Search conducted on May 22, 2021.	*n* = 24
Van Kessel et al. (2021) The Netherlands	To create an overview of the nature and frequency of persistent symptoms experienced by patients after mild COVID‐19 infection.	Sample size: *n* = 3000. Age: Mean or median range: 38–59 years. Gender: Not reported. Comorbidities: Not reported. Hospital admission: Review was focussed on data from outpatients (those with mild COVID symptoms). ICU: Not reported. *S*ocioeconomic status: Not reported.	PubMed, Embase, PsycINFO. Websites: Google scholar, Dutch College of General Practitioners (NHG) and their journal “Huisarts en Wetenschap,” Dutch journal of medicine (NTvG). Social media: Twitter.	Not reported. (The search was conducted on the February 02, 2021).	*n* = 9
Wildwing and Holt (2021) UK	To identify long‐term neurological symptoms due to COVID‐19	Sample size: 67 229 participants. Age: Not reported. Gender: Not reported. Comorbidities: Not reported. Hospital admission: Not reported. ICU: Not reported. Socioeconomic status: Not reported.	PubMed Central, Cochrane Database of Systematic Reviews, Ovid, ScienceDirect, Biomed Central, BMJ, SAGE Journals.	December 2019 to November 2020.	*n* = 45
Willi et al. (2021) Switzerland	To evaluate the available evidence of all intermediate and long‐term COVID‐19 sequelae affecting formerly healthy adults.	Sample size: *n* = 48 246. Age: Only young adults aged 18–50 years old were included in the review. Age range (of studies): 18–87 years. Gender: Female: 13%–100% Comorbidities: Not reported. Hospital admission: Not reported. ICU: Not reported. Socioeconomic status: Not reported.	Embase, PubMed, Scopus, WHO, LitCovid, bioRxiv, medRxiv.	Not reported. Search conducted between September 15, 2020 and September 17, 2020.	*n* = 31
Yusuf et al. (2021) Indonesia	To determine the cumulative prevalence of prolonged gastrointestinal symptoms, in survivors of both mild and severe COVID‐19.	Sample size: Not reported. Age: Not reported. Gender: Not reported. Comorbidities: Not reported. Hospital admission: Not reported. ICU: Not reported. Socioeconomic status: Not reported.	PubMed, Scopus, Web of Science.	2019 to January 30, 2021.	*n* = 22

Abbreviations: CFQ‐11, Chalder Fatigue Scale; CI, confidence interval; ADL, activities of living; FND, functional neurological disorder; ICU, intensive care unit; IQR, interquartile range; NCSI, Nijmegen clinical screening instrument; SARS‐CoV‐2, severe acute respiratory syndrome coronavirus 2; SF‐36, short form health survey.

**Table 3:** Quality appraisal results

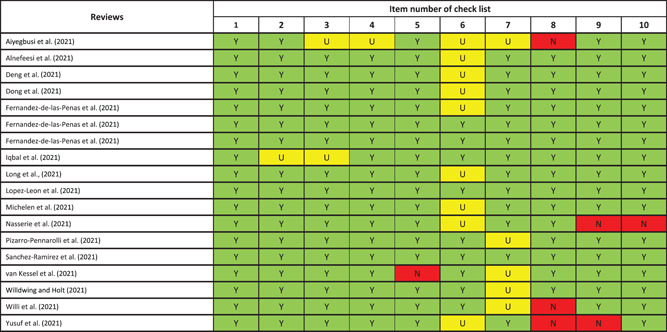

*Note*: Item number checklist key: (1) Is the review question clearly and explicitly stated, (2) Were the inclusion criteria appropriate for the review question, (3) Was the search strategy appropriate, (4) Were the sources and resources used to search for studies adequate, (5) Were the criteria for appraising studies appropriate, (6) Was critical appraisal conducted by two or more reviewers independently, (7) Were the methods used to combine studies appropriate, (8) Was the likelihood of publication bias assessed, (9) Were recommendations for policy and/or practice supported by the reported data, (10) Were the specific directives for new research appropriate. Three levels of assessment quality scores. 
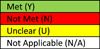

### 3.1 Holistic health impacts of post‐COVID condition

The existing evidence base is largely skewed in favor of a biomedical evaluation of health outcomes in individuals affected by post‐COVID condition, and predominately focused on physical outcomes (Table 4). In descending order of frequency, the most frequently health domain explored included: physical 15/18, psychological 10/18, cognitive 8/18, quality of life 4/18, social 3/18, health system 1/18, and spiritual 0/18.

**Table 4:** Frequency of holistic health impacts of COVID‐19 beyond acute phase

**Study**	**Physical** 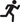	**Psychological** 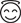	**Social** 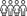	**Spiritual** 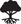	**Quality of life** 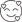	**Health system** 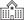	**Cognitive** 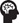	**Number of domains explored within each review**
Aiyegbusi et al. (2021)	✓	✓	‐	‐	✓	✓	✓	5
Alnefeesi et al. (2021)	‐	‐	‐	‐	‐	‐	✓	1
Deng et al. (2021)	‐	✓	‐	‐	‐	‐	‐	1
Dong et al. (2021)	‐	✓	‐	‐	‐	‐	‐	1
Fernandez‐de‐las‐Penas et al. (2021)	✓	‐	‐	‐	‐	‐	‐	1
Fernandez‐de‐las‐Penas et al. (2021)	✓	‐	‐	‐	‐	‐	‐	1
Fernandez‐de‐las‐Penas et al. (2021)	✓	‐	‐	‐	‐	‐	‐	1
Iqbal et al. (2021)	✓	‐	‐	‐	‐	‐	‐	1
Long et al. (2021)	✓	✓	‐	‐	‐	‐	✓	3
Lopez‐Leon et al. (2021)	✓	✓	‐	‐	‐	‐	✓	3
Michelen et al. (2021)	✓	✓	‐	‐	✓	‐	✓	4
Nasserie et al. (2021)	✓	✓	‐	‐	‐	‐	✓	3
Pizarro‐Pennarolli et al. (2021)	✓	✓	‐	‐	✓	‐	✓	4
Sanchez‐Ramirez et al. (2021)	✓	‐	✓	‐	✓	‐	‐	3
Van Kessel et al. (2021)	✓	✓	✓	‐	‐	‐	✓	4
Wildwing and Holt (2021)	✓	‐	‐	‐	‐	‐	‐	1
Willi et al. (2021)	✓	✓	✓	‐	‐	‐	‐	3
Yusuf et al. (2021)	✓	‐	‐	‐	‐	‐	‐	1
Number of domains explored across all reviews	15	10	3	0	4	1	8	‐

### 3.2 Physical impacts

There were a range of clinically important findings largely related to the experience of symptomatology in patients living with post‐COVID condition. Fifteen of the 18 included systematic reviews that provided information on the physical impacts. There was significant heterogeneity in the measurements used and time points of assessment which made performing a meta‐analysis problematic. Other important considerations are that the data concerning physical impacts of post‐COVID condition are limited to the frequency/prevalence of symptoms only. Symptoms are among the most common reasons that patients seek health care support but are also inter‐related with symptom intensity and bother/distress. Of the evidence which is available in relation to the physical impacts of post‐COVID condition, the most frequently reported symptoms included: fatigue, dyspnea (shortness of breath), myalgia (muscle pain), joint ache, headache, cough, chest pain, altered smell, altered taste, and diarrhea.^5^, ^11^, ^18^, ^24^, ^27^, ^29^, ^30^, ^31^, ^32^, ^33^ Less commonly reported symptoms included a runny nose, sneezing, hoarseness, and ear pain.^24^ However, it is unclear what the symptom intensity and bother/distress experiences were for these symptoms among the participants. One systematic review reported that a total of 63.2% of the sample exhibited one or more post‐COVID‐19 symptoms 30 days after onset/hospitalization, 71.9% at 60 days after, and 45.9% ≥90 days after acute onset.^29^

One systematic review^34^ focussed specifically on the overall prevalence of joint pain which was observed to be 7.7% at onset/hospital admission and 33.2%, 4.6%, 12.0%, 12.1%, at 30, 60, 90, and ≥180 days after onset/hospitalization, respectively. Of the available data, there was no significant differences between hospitalized and nonhospitalized patients.^34^ A further systematic review^35^ had the central focus of the time course of headache at onset/hospitalization to 30, 60, 90, and ≥180 days, revealed a significant effect of time (*p* < 0.001) showing that the prevalence of headache dropped from the symptoms' onset to all post‐COVID‐19 follow‐up periods and was maintained afterwards.^35^

Two reviews explored factors which predicted the experience of post‐COVID condition physical symptoms. One systematic review identified that hospitalization, and age between 40 and 49 years, were the two most significant predictors of post‐COVID condition.^30^ However other factors including the initial presentation symptoms (fever, dyspnea, anosmia, ageusia, and chest pain), gender or the number of comorbidities, did not predict post‐COVID condition.^30^ In contrast, a different systematic review reported that comorbidities, increasing age, being female, a loss of taste, and minority ethnicity were associated with post‐COVID condition.^18^ Therefore, the evidence about physical and demographic predictors of post‐COVID condition is conflicting at this stage.

### 3.3 Psychological impacts

Ten systematic reviews^5^, ^18^, ^24^, ^26^, ^31^, ^32^, ^33^, ^36^, ^37^, ^38^ identified the psychological impacts of post‐COVID condition which included anxiety, depression, posttraumatic stress disorder, somatization, fear, attention deficit disorder, and hair loss. Hair loss was thought to be more psychosocial in nature rather than caused by physical consequences of COVID‐19^31^ due to emotional distress which lasted up‐to 3 months postacute COVID‐19.^5^ Participants also reported thoughts of self‐harm and suicidal tendencies.^24^ The most commonly experienced psychological impacts included depression 45% (*n* = 4028, 95% CI: 37%–54%), anxiety 47% (*n* = 3315, 95% CI: 37%–57%) and sleep disturbances 34% (*n* = 1795, 95% CI: 19%–50%) but these were not associated with gender or age.^36^ Another review reported that severity of the infection was associated with different levels of anxiety, depression and posttraumatic stress, somatization, and fear.^37^ Some participants expressed concerns that physical and psychological recovery was not possible and this caused them distress.^33^ Other participants described a change in their identity when they reflected on how they perceived themselves before being diagnosed with COVID‐19.^33^ One systematic review reported an increased incidence of 5.8% of newly diagnosed psychiatric diseases 14–90 days after diagnosis of COVID‐19 infection.^26^

### 3.4 Cognitive impacts

Negative cognitive impacts were also commonly reported across eight systematic reviews.^5^, ^18^, ^24^, ^31^, ^32^, ^33^, ^38^, ^39^ Participants reported problems with cognition likened to “brain fog,” amnesia (memory loss) and difficulties concentrating.^24^, ^32^ The prevalence of cognitive impairments ranged from 26%,^18^ 35%,^31^ and 43% to 66.8%.^32^, ^39^ One systematic review identified that negative cognitive impairments were persistent at 13 weeks postacute disease onset.^33^

### 3.5 Quality of life

The impact of post‐COVID condition on quality of life was infrequently reported across the included systematic reviews. Four systematic reviews reported that individuals affected by post‐COVID condition reported reduced quality of life.^11^, ^18^, ^24^, ^38^ One review identified that participants self‐reported a clinically significant decrease in quality of life on average 48 ± 10.3 days postacute phase, which impacted reduced mobility, ability to self‐care, participation in usual activities, pain/discomfort, and anxiety/depression as measured by the EuroQol intrument.^38^

### 3.6 Social impacts

Three systematic reviews identified that individuals affected by post‐COVID condition experienced some problems with returning to work/employment at ∼3 months postacute phase.^11^, ^33^ A separate systematic review identified that patients affected by post‐COVID condition reported a loss of income and ability to work secondary to fatigue.^33^ Cognitive impairments and fatigue limited the prospect of individuals affected by post‐COVID condition to find new employment or return to work.^11^ It was estimated that 31% could not return to employment at 72 days postacute hospital discharge.^26^

### 3.7 Health system impacts on health

Only one review provided insight into the impact of the health system on recovery for individuals grappling with post‐COVID condition.^24^ People living with post‐COVID condition reported that they felt a sense of “abandonment” or were being “dismissed” by healthcare providers and received limited or conflicting advice to inform their rehabilitation and self‐management.

### 3.8 Spiritual impacts

None of the reviews provided any information on the spiritual impacts of living with post‐COVID condition.

## 4 DISCUSSION

The main finding of this umbrella review, capturing almost 300 000 participants with post‐COVID condition, is that there is an enormous breadth of challenges (physical, psychological, and cognitive) impacting on quality of life and social participation beyond the acute phase of the illness. Acknowledging the heterogeneity of symptoms between presentations, frequently experienced problems in people with post‐COVID condition include physical symptoms (fatigue, dyspnea, myalgia); psychological issues (depression, anxiety, sleep disturbance); and cognitive deficits (“brain fog,” amnesia, difficulty concentrating). There were no consistent physical or demographic predictors of any of these symptom clusters across the different reviews, and such symptoms were found in people whether or not they had been hospitalized with COVID‐19. The breadth of symptomatology, variable time course, and absence of clear predictors of those at risk, renders treatment of people with post‐COVID condition particularly challenging.

The evidence regarding impact of post‐COVID condition on social participation is less clear, but it appears that physical weakness, cognitive impairments, and fatigue can make it difficult for approximately a quarter of people to return to work for 2 to 3 months following COVID‐19 infection. The wider social impact of workforce limitations is beyond the scope of this review but deserves further analysis with respect to workforce planning in the coming years.

The limited evidence regarding the impact of the health system on the recovery trajectory is not favorable, and conflicting advice about rehabilitation and self‐management might further exacerbate the challenges of recovery from post‐COVID condition. Meanwhile, the complete lack of evidence regarding the spiritual impacts of COVID‐19 suggests that health care providers and society more broadly are not yet exploring a truly holistic model of assessment and treatment for people living with post‐COVID condition.

The challenges of prolonged recovery from a critical illness are not new.^40^ It is possible that the problems of COVID‐19 survivorship are not directly a function of the underlying pathophysiology of a specific virus, as much as the nature of recovery from any major multisystem illness. A recent case‐control study of patients admitted to the ICU with COVID‐19 found that while the onset of new functional disability at 6 months following diagnosis was high (39%),^41^ this was comparable to a matched intensive care cohort without COVID‐19.^42^ In this context, it may be prudent to focus future research efforts on improving recovery services catering to all survivors of critical illness, rather than COVID‐19 as a unique case. Alternatively, services designed to meet the needs of people with post‐COVID condition might be appropriate for much broader cohorts of survivors of critical illness in future.

There are clear deficits in our understanding of predictors of post‐COVID condition. Until we have more data, health care providers should ensure meticulous screening and education of people with COVID‐19 to identify those struggling with ongoing symptoms for months beyond their COVID‐19 diagnosis and link them with appropriate support services. Given the data summarized in this umbrella review, such services might include nursing, physiotherapy, occupational therapy, psychology, and more; however, given the known heterogeneity of symptom burden, multidisciplinary clinics may be best placed to provide comprehensive individually tailored interventions. In Australia, there is a dearth of such clinics with only two ICU follow‐up clinics noted in a recent survey.^43^ However, the prevalence of COVID‐19 may accelerate proliferation of such clinics to enhance patients' recovery in the future. The evidence to support the efficacy of multidisciplinary clinics is a much‐needed future research direction.

The evidence summarized in this review is highly skewed towards a biomedical evaluation of health outcomes in individuals affected by post‐COVID condition. Furthermore, none of the incorporated reviews provide any information on symptom intensity and bother/distress or symptom clusters. These are important clinical considerations and gaps in the existing evidence base. The strong focus on physical outcomes underscores a lack of information about the holistic health impacts to inform person‐centered models of rehabilitation. Given the complexity and variability of presentation, a person‐centered approach is needed for those affected by post‐COVID condition to meet their informational, spiritual, psychological, social, and physical needs. There is clearly still much work to do in adequately assessing these domains in people with post‐COVID condition, let alone providing effective and appropriate holistic interventions.

## 5 LIMITATIONS

Limitations in the data set captured by this umbrella review include a lack of research in non‐WEIRD^28^ populations; incomplete data sets that did not always capture key details (such as whether participants required admission to hospital or intensive care); and an inability to analyse the data with respect to different variants of COVID‐19. The impact of vaccination programs on the manifestations of post‐COVID condition cannot be gleaned from this data, and future studies should re‐examine the presentation of post‐COVID condition in populations with high vaccination rates to ascertain whether the conclusions drawn in this review remain valid in such cohorts.

## 6 CONCLUSION

Acute COVID‐19 has impacted millions across the world. The long term sequalae of acute COVID‐19 and size of the problem is only beginning to emerge, which has not only an impact upon the physical and mental well‐being of individuals and their carers but also upon the health care system and the community. Further investigation is needed to understand the broader range of factors that predict or influence the presence and manifestation of post‐COVID condition. Further, those affected by post‐COVID condition must have their informational, spiritual, psychological, social, and physical needs met, and personalized approaches developed that support people in their recovery journey.

## AUTHOR CONTRIBUTIONS

**Catherine Paterson**: Protocol development; reviewer screening; data extraction; quality assessment; paper writing. **Deborah Davis**: Protocol development; reviewer screening; manuscript editing. **Michael Roche**: Review screening; manuscript editing. **Bernie Bissett**: Protocol development; reviewer screening; paper writing. **Cara Roberts**: Protocol development; reviewer screening; data extraction; quality assessment; paper writing. **Murray Turner**: Database searches; manuscript editing. **Imogen Mitchel**: Manuscript editing. **Emma Baldock**: Consumer representative.

## CONFLICT OF INTEREST

The authors declare no conflict of interest.

## Supporting information

Supporting information.

## Data Availability

Not applicable for this systematic review.
