# Supplementary material for: What are the long‐term holistic health consequences of COVID‐19 among survivors? An umbrella systematic review
Source: J Med Virol. 2022 Sep 3:10.1002/jmv.28086. Online ahead of print. doi: 10.1002/jmv.28086 (PMC9539336; doi:10.1002/jmv.28086)
Supplement: Supplementary file 1 — Supporting information. [file JMV-9999-0-s001.docx]

**Supplementary Table 1. Database searches**

| Database: CINAHL on EBSCOhost | | | |
| --- | --- | --- | --- |
| Date of search: 8/10/2021 | | | |
| Symbols used in this document: | | | |
| MH = Main Heading or “CINAHL Heading” | | | |
| Asterisk * = Truncator – finds various endings of a word stem | | | |
| N3 = Proximity – Finds words located no more than 3 words proximal | | | |
| **Search #** | **Concept/Explanation** | **Search Terms/Strategy** | **# of Results** |
| #1 | Sample (S): People affected by long-term consequences of COVID-19. | ((MH "COVID-19") OR (MH "SARS-CoV-2") OR covid-19 OR coronavirus OR 2019-ncov OR sars-cov-2 OR cov-19) | 71,784 |
| #2 | Phenomena of Interest (PI): The physical, psychological, social, and spiritual impacts on health among COVID-19 survivors. | ((emotion* OR health OR mental OR physical OR psychol* OR quality-of-life OR social OR spiritual OR wellbeing OR well-being) N3 (consequenc* OR effect* OR impact* OR influence* OR outcome*)) | 1,054,133 |
| #3 | Design (D): All qualitative, quantitative, and mixed methods reviews. | critical-review OR descriptive-review OR evidence-synthesis OR literature-review OR meta-analysis OR mixed-methods-review OR narrative-review OR qualitative-review OR rapid-review OR realist-review OR review-of-reviews OR scoping-review OR systematic-review OR umbrella-review | 245,288 |
| #4 | Qualitative, quantitative, and mixed methods reviews about physical, psychological, social, and spiritual impacts on health among people affected by long-term consequences of COVID-19. | #1 AND #2 AND #3 | 794 |
|  | Language English | Limiter applied | 767 |

| Database: MEDLINE on EBSCOhost | | | |
| --- | --- | --- | --- |
| Date of search: 8/10/2021 | | | |
| Symbols used in this document: | | | |
| MH = Main Heading or “MeSH Heading” | | | |
| Asterisk * = Truncator – finds various endings of a word stem | | | |
| N3 = Proximity – Finds words located no more than 3 words proximal | | | |
| **Search #** | **Concept/Explanation** | **Search Terms/Strategy** | **# of Results** |
| #1 | Sample (S): People affected by long-term consequences of COVID-19. | ((MH "COVID-19") OR (MH "SARS-CoV-2") OR covid-19 OR coronavirus OR 2019-ncov OR sars-cov-2 OR cov-19) | 195,505 |
| #2 | Phenomena of Interest (PI): The physical, psychological, social, and spiritual impacts on health among COVID-19 survivors. | ((emotion* OR health OR mental OR physical OR psychol* OR quality-of-life OR social OR spiritual OR wellbeing OR well-being) N3 (consequenc* OR effect* OR impact* OR influence* OR outcome*)) | 3,550,711 |
| #3 | Design (D): All qualitative, quantitative, and mixed methods reviews. | critical-review OR descriptive-review OR evidence-synthesis OR literature-review OR meta-analysis OR mixed-methods-review OR narrative-review OR qualitative-review OR rapid-review OR realist-review OR review-of-reviews OR scoping-review OR systematic-review OR umbrella-review | 477,236 |
| #4 | Qualitative, quantitative, and mixed methods reviews about physical, psychological, social, and spiritual impacts on health among people affected by long-term consequences of COVID-19. | #1 AND #2 AND #3 | 3,556 |
|  | Language English | Limiter applied | 3,493 |

| Database: PsycINFO on EBSCOhost | | | |
| --- | --- | --- | --- |
| Date of search: 8/10/2021 | | | |
| Symbols used in this document: | | | |
| Asterisk * = Truncator – finds various endings of a word stem | | | |
| N3 = Proximity – Finds words located no more than 3 words proximal | | | |
| **Search #** | **Concept/Explanation** | **Search Terms/Strategy** | **# of Results** |
| #1 | Sample (S): People affected by long-term consequences of COVID-19. | covid-19 OR coronavirus OR 2019-ncov OR sars-cov-2 OR cov-19 | 10,159 |
| #2 | Phenomena of Interest (PI): The physical, psychological, social, and spiritual impacts on health among COVID-19 survivors. | ((emotion* OR health OR mental OR physical OR psychol* OR quality-of-life OR social OR spiritual OR wellbeing OR well-being) N3 (consequenc* OR effect* OR impact* OR influence* OR outcome*)) | 1,868,375 |
| #3 | Design (D): All qualitative, quantitative, and mixed methods reviews. | critical-review OR descriptive-review OR evidence-synthesis OR literature-review OR meta-analysis OR mixed-methods-review OR narrative-review OR qualitative-review OR rapid-review OR realist-review OR review-of-reviews OR scoping-review OR systematic-review OR umbrella-review | 123,599 |
| #4 | Qualitative, quantitative, and mixed methods reviews about physical, psychological, social, and spiritual impacts on health among people affected by long-term consequences of COVID-19. | #1 AND #2 AND #3 | 255 |
|  | Language English | Limiter applied | 243 |
| Database: Scopus | | | |
| Date of search: 8/10/2021 | | | |
| Symbols used in this document: | | | |
| Asterisk * = Truncator – finds various endings of a word stem | | | |
| W/3 = Proximity – Finds words located no more than 3 words proximal | | | |
| **Search #** | **Concept/Explanation** | **Search Terms/Strategy** | **# of Results** |
| #1 | Sample (S): People affected by long-term consequences of COVID-19. | covid-19 OR coronavirus OR 2019-ncov OR sars-cov-2 OR cov-19 | 248,807 |
| #2 | Phenomena of Interest (PI): The physical, psychological, social, and spiritual impacts on health among COVID-19 survivors. | ((emotion* OR health OR mental OR physical OR psychol* OR quality-of-life OR social OR spiritual OR wellbeing OR well-being) W/3 (consequenc* OR effect* OR impact* OR influence* OR outcome*)) | 1,009,183 |
| #3 | Design (D): All qualitative, quantitative, and mixed methods reviews. | critical-review OR descriptive-review OR evidence-synthesis OR literature-review OR meta-analysis OR mixed-methods-review OR narrative-review OR qualitative-review OR rapid-review OR realist-review OR review-of-reviews OR scoping-review OR systematic-review OR umbrella-review | 780,548 |
| #4 | Qualitative, quantitative, and mixed methods reviews about physical, psychological, social, and spiritual impacts on health among people affected by long-term consequences of COVID-19. | #1 AND #2 AND #3 | 930 |
|  | Language English | Limiter applied | 895 |
